# Geometric deep learning as a potential tool for antimicrobial peptide prediction

**DOI:** 10.3389/fbinf.2023.1216362

**Published:** 2023-07-13

**Authors:** Fabiano C. Fernandes, Marlon H. Cardoso, Abel Gil-Ley, Lívia V. Luchi, Maria G. L. da Silva, Maria L. R. Macedo, Cesar de la Fuente-Nunez, Octavio L. Franco

**Affiliations:** ^1^ Centro de Análises Proteômicas e Bioquímicas, Pós-Graduação em Ciências Genômicas e Biotecnologia, Universidade Católica de Brasília, Brasília, Brazil; ^2^ Departamento de Ciência da Computação, Instituto Federal de Brasília, Brasília, Brazil; ^3^ S-Inova Biotech, Programa de Pós-Graduação em Biotecnologia, Universidade Católica Dom Bosco, Campo Grande, Brazil; ^4^ Laboratório de Purificação de Proteínas e suas Funções Biológicas, Universidade Federal de Mato Grosso do Sul, Cidade Universitária, Campo Grande, Mato Grosso do Sul, Brazil; ^5^ Machine Biology Group, Departments of Psychiatry and Microbiology, Perelman School of Medicine, Institute for Biomedical Informatics, Institute for Translational Medicine and Therapeutics, University of Pennsylvania, Philadelphia, PA, United States; ^6^ Departments of Bioengineering and Chemical and Biomolecular Engineering, School of Engineering and Applied Science, University of Pennsylvania, Philadelphia, PA, United States; ^7^ Penn Institute for Computational Science, University of Pennsylvania, Philadelphia, PA, United States

**Keywords:** antimicrobial peptide prediction, geometric deep learning, antimicrobial peptide classification, antimicrobial peptide design, explainable artificial intelligence

## Abstract

Antimicrobial peptides (AMPs) are components of natural immunity against invading pathogens. They are polymers that fold into a variety of three-dimensional structures, enabling their function, with an underlying sequence that is best represented in a non-flat space. The structural data of AMPs exhibits non-Euclidean characteristics, which means that certain properties, e.g., differential manifolds, common system of coordinates, vector space structure, or translation-equivariance, along with basic operations like convolution, in non-Euclidean space are not distinctly established. Geometric deep learning (GDL) refers to a category of machine learning methods that utilize deep neural models to process and analyze data in non-Euclidean settings, such as graphs and manifolds. This emerging field seeks to expand the use of structured models to these domains. This review provides a detailed summary of the latest developments in designing and predicting AMPs utilizing GDL techniques and also discusses both current research gaps and future directions in the field.

## Introduction

Bacterial resistance to antibiotics is causing a rise in mortality due to what were once treatable infections. Novel strategies to counter such infections are needed. AMPs have been recognized as promising substitutes for traditional therapies (Huemer et al, 2020; Magana et al, 2020). These bioactive peptides display a low molecular mass and often possess high antimicrobial, antibiofilm, and anti-inflammatory activities, in addition to encouraging toxicity profiles (de la Fuente-Nunez et al, 2016; Silva et al, 2016). This class of antimicrobials is also less likely than conventional antibiotics to select for bacterial resistance (Luo and Song, 2021). AMPs have a net-positive charge that can interact with the bacterium’s net-negatively charged membrane through two primary mechanisms of action: the peptide can either interfere with the cell membrane causing lysis or penetrate the membrane to compromise bacterial metabolism, among other intracellular targets, eventually leading to cell death (Lazzaro et al, 2020).

In order to design a novel AMP candidate, its physicochemical properties, structural profile, and biological activities, especially its specific molecular targets, must be well elucidated. To become a therapeutic candidate, the peptide must also have bioavailability in the organism; in particular, it must be stable in human plasma. Furthermore, to be safely administered in the human body, the peptide must exhibit both high affinity and specificity towards the target it is meant to bind to (Wang et al, 2022). All these properties have been used experimentally for subsequent AMP prediction. However, the *in vitro* experiments required for collecting such parameters are usually laborious, expensive, and time-consuming (Chung et al, 2020). Consequently, computational methods have emerged as exciting avenues for precise AMP discovery and rational design (Xu et al, 2021).

Numerous AMPs are now available in publicly accessible databases, partly due to progress enabled by computational methods, which are valuable resources for recognizing AMP patterns and determinants that are crucial for biological function (Singh et al, 2016; Wang et al, 2016; Porto et al, 2018; de la Fuente-Nunez, 2019; Waghu and Idicula-Thomas, 2020; Yan et al, 2020; Palmer et al, 2021; Torres et al, 2021; Torres et al, 2022; Wan et al, 2022). Thus, algorithms have been developed that can learn from previously provided data and solve problems related to this learned information (Melo et al, 2021). Machine learning procedures, including (RF) random forest, have gained significant popularity in the prediction of therapeutic drugs (Manne and Kantheti, 2021; Söylemez et al, 2022). They have been fruitfully applied for proteome-wide cleavage (PWC) site prediction, establishing paleoproteome mining as a methodological approach to identify novel peptide antibiotics (Maasch et al, 2022) and for accurately predict putative AMP against Gram-negative and positive bacteria (Söylemez et al, 2022).

AMP prediction methods based on deep learning have demonstrated advantages over other computational tools. Deep learning approaches can collect and integrate a large amount of information in a nonlinear way, getting more connections between the data points and, therefore, gathering more knowledge (Gupta et al, 2021). Increasing developments in deep learning methods, such as deep generative models, have increased the reliability of prediction and generation of AMPs. Deep generative models have produced promising peptides by (1) assigning an AMP probability from the data distribution, (2) generating novel AMPs that possess properties similar to those AMPs present in the training data, and (3) extracting expressive data representations or executing casual inference by specifying the AMP generation process. Large language models such as long-short term memory (LSTM) and a bidirectional LSTM have been effectively constructed to design novel AMP molecules against *E. coli.* (Wang et al, 2021)*.* Deep generative models have been successful in producing promising results for the creation of novel drug-like molecules, including the identification of potential antimicrobial peptides that can be prioritized for further wet-lab experimentation (Rossetto and Wenjin, 2020; Li et al, 2022; Wan et al, 2022; Zhang et al, 2023).

**FIGURE 1:**
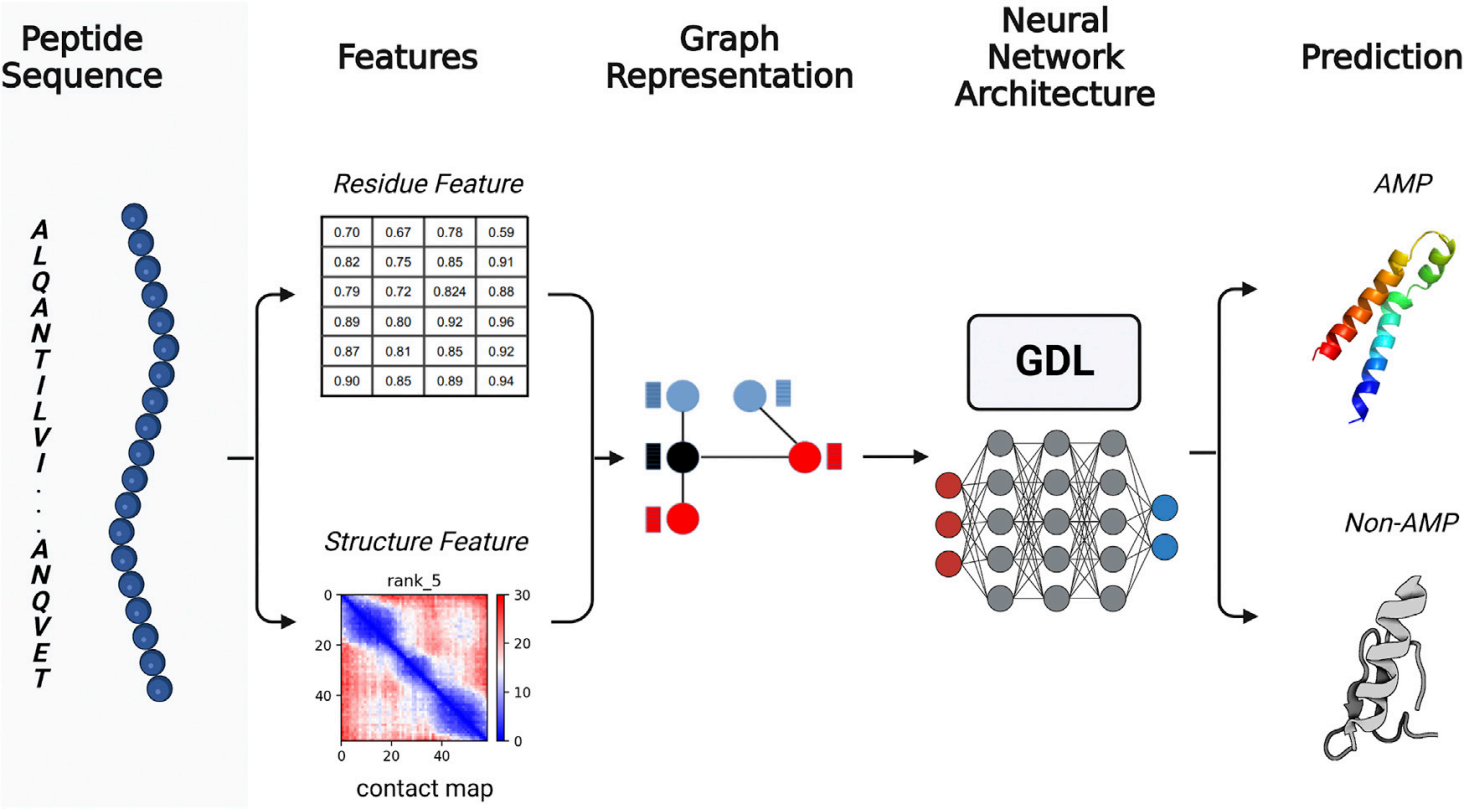
General rational pipeline for antimicrobial peptide (AMP) prediction using GDL. From an initial putative AMP amino acid sequence, the relevant physicochemical characteristics are extracted, and the three-dimensional structure of the sequence is predicted. Once the sequence and spatial relationships are obtained, they are converted into graphs in which the structural information is represented by the edges, while the amino acid residue information is represented by the nodes. The graph-based data is presented to a GDL network to predict whether the candidate is likely to have antimicrobial activity. Created with BioRender.com.

Among deep learning methods leveraged for AMP predictions, the most widely used are convolutional neural networks (CNNs) (Li et al, 2021). The CNNs used in conventional deep learning assume that the data are related and organized as a regular grid, following the parameters of Euclidean geometry (Li et al, 2021). Nonetheless, the three-dimensional structure of peptides and proteins is better represented in a non-Euclidean space because its manifold data cannot be flattened without significant distortions. To implement deep learning for prediction in non-Euclidean systems, geometric deep learning emerges as a more efficient computational tool compared to several advanced and contemporary techniques. This is due to the fact that geometric deep learning can properly recognize and decipher the biochemical and geometric patterns of a given molecule (Rao et al, 2020; Yan K. et al, 2022; Puentes et al, 2022; Sun et al, 2022). Since geometric deep learning shows promise when applied to AMP prediction, it is the main focus of this review article (Huemer et al, 2020; Gainza et al, 2020) (Figure 1).

## Geometric deep learning for AMP prediction

To improve the representation of the three-dimensional structure and physicochemical properties of amino acids, AMPs can be modeled as graphs that are based on either their structural data or manifolds describing their geometric shapes. Considering the small size of AMPs compared to proteins, the burden of having a graph with a large number of nodes representing the data size reduces the challenge for machine learning processing.

Distinctive geometric deep learning methods for graphs have been proposed thus far for general applications (i.e., image and signal processing, traffic flow forecast, recommender systems, natural language processing, etc.), such as spectral-based graph convolutional networks: spectral convolutional neural networks (SCNN) are based on the application of the Fourier transform to graphs (Bruna et al, 2013); smooth SCNN use filters that are spatially localized in the frequency domain (Henaff et al, 2015); Chebyshev spectral CNN (ChebNet) applies the Chebyshev polynomial basis to represent the filters of spectral CNNs (Defferrard et al, 2016); graph convolutional networks (GCN) employs filters that process the graph’s one-hop neighborhoods (Kipf and Welling, 2016); adaptive graph convolutional networks (AGCNs) use a residual graph that is formed by computing the pairwise distance between nodes as the graph is expanded. (Li et al, 2018); and GCN with complex rational spectral filters (CayleyNets) uses the parametric rational complex function (Levie et al, 2019).

Spatial-based graph convolutional networks, such as graph neural networks (GNN), have also been proposed. Until they reach a state of convergence, they repeatedly adjust and improve the hidden representation of nodes (Scarselli et al, 2009). GraphSage, as an instance, employs an aggregation function to define the spatial domain convolution on a graph (Hamilton et al, 2017). The Diffusion CNN (DCNN) utilizes a random walk procedure on the graph. (Atwood and Towsley, 2016), Patchy-San approach involves transforming structural data of a labeling graph into a structural grid, and then applying a CNN to handle graph classification tasks in a shift-invariant manner (Niepert et al, 2016), with large-scale graph convolutional networks (LGCN) suggest a sorting technique that relies on the information present in the feature of nodes (Gao et al, 2018), and mixture model networks (MoNet) expand the CNN structure to domains that are non-Euclidean (Monti et al, 2017). In graph attention neural networks (GAT), attention mechanisms are applied to evaluate the significance of each neighboring node (Vaswani et al, 2017). On the other hand, graph generative networks (GGN) create a new graph from a specified collection of observed graphs based on a given sentence (Chen et al, 2018), and graph auto-encoders (GAE) use neural network architecture to transform network vertices into a vector space with fewer dimensions (Kipf and Welling, 2016). Some of the graph methods mentioned above have already been applied to AMP prediction and have outperformed current methods based on Euclidean space (Table 1) (Cao et al, 2020).

**TABLE 1:** Summary of AMP prediction approaches using GDL methods.

*Predictor’s name*	*Applied prediction method*	*Outperforms*	*Paper Reference*
sAMPpred-GAT	Graph Attention Neural Networks (GAT)	amPEPpy, AMPfun, AMPEP, ADAM-HMM, Ampir, AMPScannerV2, AmpGram, Deep-AMPEP30a, CAMP-ANN	Yan et al (2022b)
AMPs-Net	GCN	AMPScanner, AI4AMPs, CAMPR3, AMPDiscover, AMPlify, AMPEPpy (RF)	Puentes et al (2022)
LABAMPsGCN	GCN and Chebyshev Spectral CNN	CAMP-SVM, iAMP-2L, AMPfun	Sun et al (2022)
ACP-GCN	GCN	Convolutional neural network, long short-term memory (outperforms for accuracy)	Rao et al (2020)

Yan K. et al (2022) established the sAMPpred-GAT that captures characteristics at the amino acid residue level by incorporating sequence information and spatial interrelationships among residues that are obtained from predicted protein structures. To integrate peptide information, graphs are constructed containing edges, which represent structural information, and nodes, which represent sequence information and evolutionary information. Next, a GAT is employed to derive characteristics from the data presented in a graph format, followed by the use of a linear layer to determine if a given peptide exhibits antimicrobial properties. The method comprises four comprehensive features: one-hot encoding, position encoding, position-specific scoring matrices, and hidden Markov models. To predict the structure of a protein, the contact map of the predicted protein structure is utilized to obtain the distance and angle measurements for each pair of amino acid residues. To make predictions, graphs are created using both structural and sequence attributes, and a neural network that employs a GAT is used to integrate the data from adjacent nodes. The final layers use the graph-level context vector to forecast if the peptide possesses antimicrobial activity or not. The findings indicate that sAMPpred-GAT surpasses alternative approaches, demonstrating superior or closely comparable outcomes in eight distinct test datasets, as assessed by the area under the curve (AuC). sAMPpred-GAT achieved superior performance, as measured by the area under the curve (AuC), Matthews correlation coefficient (MCC), accuracy, sensitivity, and specificity, by leveraging two types of information: (1) features obtained from the graph-based data produced using amino acid characteristics from sequence information, and (2) spatial relationships derived from the predicted structural information. This approach outperforms most of the current cutting-edge methods. (Yan K. et al, 2022).

AMPs-Net, as presented by Puentes et al (2022), involves the conversion of peptide sequences into graph representations, where nodes match to edges and atoms corresponding to bonds. Nine physicochemical properties are used to represent each amino acid, and bonds between the amino acid residues are described by three properties: type (single, double, triple and aromatic, stereochemistry (none, z, e, cis, trans, any), and conjugation (true or false). The GCN module is a message-passing approach, which has been employed to forecast the characteristics or attributes of peptide graphs at the molecular level. The GCN module comprises 20 message-passing layers, utilized softmax as its aggregation function, and employed a four-layer MLP as its update function. The resulting graph contained 256 feature vectors for each amino acid residue and bond. To generate a single representation for each peptide, average pooling was utilized. The metadata vector (comprising eight peptide physicochemical properties) was merged with this representation and then inputted into a linear layer to generate a new vector. This vector was then applied for binary and multiclass classification, predicting AMP, and evaluating the probabilities of AMP activity. Moreover, method outclassed four other deep learning methods, demonstrating an improvement of 8.80%–19.02% in average precision and 5.74%–24.23% in accuracy (Puentes et al, 2022).

Sun et al (2022) developed a GCN to predict lactic acid bacteria AMPs (LABAMPs). This model employed a vast, diverse graph based on amino acid sequences and peptides, encompassing amino acids, dipeptides, and tripeptides. The peptides were represented as words (segmentation of an amino acid sequence), after filtering and counting, to acquire the words that are needed to function as nodes in a graph. The edges can connect nodes of peptide segments or nodes of peptide segments and sequences. The word embedding co-occurrence technique is used to obtain heterogeneous graphs representing sequence nodes and word nodes. An adjacency matrix was computed to represent the peptide information on the graph by means of its edges. In the subsequent stage, each word was incorporated using one-hot embedding and sent along with the sequence for model training. Finally, a GCN acquired knowledge regarding the connections between nodes on the graph and transmitted the pertinent details, guided by labels, to attain node classification. After 10-fold cross-validation on two different training datasets, the LABAMPs model presented an accuracy of 0.9163 and 0.9379. For independent testing datasets, the model achieved an accuracy of 0.9130 and 0.9291, outperforming other machine learning algorithms (Sun et al, 2022).

Rao et al (2020) proposed a new GCN learning-based computational model to detect anticancer peptides. The one-hot encoding technique extracted the features from the peptide amino acid sequence to construct an adjacency matrix and the amino acid graph representation. The graph edges were built by using the peptide co-occurrence information. To optimize the classification outcome, the cross-entropy metric was used as the loss function. This proposed model outperformed commonly used neural network methods, such as CNN and CNN-LSTM (Rao et al, 2020).

## Explainable artificial intelligence for AMP design

Explainable artificial intelligence (XAI) has emerged as a remarkable tool to enhance the accuracy and understanding of machine learning approaches as applied to drug design (Jimenez-Luna et al, 2020). XAI aims to provide a transparent rationale for AMP predictions made by machine learning models whose input data is not interpretable, and the output is usually regarded as a black box outcome because due to its high dimensionality and non-linear nature. The complex combination of physicochemical, structural, and compositional properties of amino acids as input to machine learning systems is still a limiting factor for interpretations with XAI (Yan J. et al, 2022). By enabling researchers to generate accurate predictions and explanations of the underlying mechanisms involved in AMP-bacteria interactions, XAI can help accelerate the discovery and development of new pharmaceutically active molecules (Preuer et al, 2019; Jimenez-Luna et al, 2020).

## Conclusion

GDL has achieved promising accuracy levels for predicting AMPs; additional methods not yet applied in this area, such as GAE, GGN, MoNet, GNN, SCNN, etc., promise to further improve performance. Future work should focus on furthering our understanding of how machine learning models are able to predict molecular function. XAI methods have been applied to drug design and protein-ligand interactions with some success but not yet to AMP design. Although several limitations still need to be overcome, GDL methods hold great promise for antimicrobial peptide prediction and design.

The GDL techniques application in the AMP domain will result in better AMP structure modeling and further functional relation understanding due to its non-Euclidean nature. Furthermore, more accurate AMP prediction and rational design of new targeted specific molecules. Ultimately, the manifold AMP representation can create a new corpus of peptide language that can be used for large language models and improve the drug design process.
